# Trends in Swedish physicians’ attitudes towards physician-assisted suicide: a cross-sectional study

**DOI:** 10.1186/s12910-021-00652-0

**Published:** 2021-07-02

**Authors:** Niels Lynøe, Anna Lindblad, Ingemar Engström, Mikael Sandlund, Niklas Juth

**Affiliations:** 1 Centre for Healthcare Ethics, Karolinska Institute, 171 77 Stockholm, Sweden; 2 University Health Care Research Centre, Faculty of Medicine and Health, Örebro University, Örebro, Sweden; 3 Department of Clinical Science/Psychiatry, Umeå University, 90185 Umeå, Sweden

**Keywords:** Physician assisted suicide, Physician attitude, Prescribing drugs, Trust in healthcare, Non-maleficent principle, Autonomy principle

## Abstract

**Aims:**

To examine attitudes towards physician-assisted suicide (PAS) among physicians in Sweden and compare these with the results from a similar cross-sectional study performed in 2007.

**Participants:**

A random selection of 250 physicians from each of six specialties (general practice, geriatrics, internal medicine, oncology, surgery and psychiatry) and all 127 palliative care physicians in Sweden were invited to participate in this study.

**Setting:**

A postal questionnaire commissioned by the Swedish Medical Society in collaboration with Karolinska Institute in Stockholm.

**Results:**

The total response rate was 59.2%. Slightly fewer than half [47.1% (95% CI 43.7–50.5)] of the respondents from the six specialties accepted PAS, which is significantly more than accepted PAS in the 2007 study [34.9% (95% CI 31.5–38.3)]. Thirty-three percent of respondents were prepared to prescribe the needed drugs. When asked what would happen to the respondent’s own trust in healthcare, a majority [67.1% (95% CI 63.9–70.3)] stated that legalizing PAS would either not influence their own trust in healthcare, or that their trust would increase. This number is an increase compared to the 2007 survey, when just over half [51.9% (95% CI 48.0–55.2)] indicated that their own trust would either not be influenced, or would increase.

**Conclusions:**

The study reveals a shift towards a more accepting attitude concerning PAS among physicians in Sweden. Only a minority of the respondents stated that they were against PAS, and a considerable proportion reported being prepared to prescribe the needed drugs for patient self-administration if PAS were legalized.

## Introduction

In recent years, there has been a lively public debate about physician-assisted suicide (PAS) both internationally and in Sweden [1–11]. California and other states in the US have followed Oregon in legalization of PAS; Canada has legalized euthanasia and PAS, and in 2020, New Zealand passed legislation laying the groundwork for the legalization of euthanasia and/or PAS in 2021. In Europe; Belgium, Holland, and Luxembourg have legalized or accepted euthanasia and PAS, and parliaments in Portugal and Spain have passed laws to begin legalization. In Sweden, proponents of PAS have called for a parliamentary inquiry into its legalization, while opponents have highlighted risks and pitfalls. At present, the legal status of PAS in Sweden is still unclear and a healthcare professional involved in PAS would probably risk losing their license to practice; however, this issue has yet to be decided in court. In the last decade, a number of patients from Sweden have travelled to Switzerland (where PAS is, in effect, legal) in order to end their lives through PAS. Several people who have been engaged in the public debate about legalizing PAS in Sweden have, in order to raise awareness about the issue, planned trips to Switzerland for the procedure [8].

A study we performed in 2007 (published in 2008) revealed that approximately 34% of Swedish physicians were in favour of PAS, given certain conditions [11]. This number is quite a bit lower than the general public, where 73% were in favour of PAS in the same year [12], and a very recent opinion poll (2020) among the general public resulted in similar numbers in favour of PAS [13]. That healthcare professionals are more against PAS than the general public is a pattern that has been documented in many countries where PAS is not legal [1, 2, 9, 10].

An important part of the public discussion in Sweden is whether PAS is a decision for citizens to make for themselves, or whether it is a decision that physicians must make, both professionally (that is, whether any physician should be involved in patient suicide) and on a case-by-case basis (that is, when it comes evaluating whether an individual patient is a candidate for PAS). It is important to point out that in Sweden there is no legal room for a physician to conscientiously object to PAS or any other procedure [14]. Regardless of who is responsible for the decision, it is in the interest of society to know how physicians view the issue of PAS, and whether the trends in attitudes seen in other countries (for example, more accepting attitudes towards PAS have been reported in the UK (1) and Finland (2) over time) may also be observed in Sweden.

The current study is a follow-up to the study we conducted 2007, with the aim of examining trends and comparing current attitudes towards PAS among physicians in Sweden [11]. The reason for limiting the study to questions about PAS is that the public and professional debate in Sweden has exclusively been about PAS, and not for example about euthanasia.

## Methods and participants

### Participants

A randomized selection of 250 physicians from each of six specialties (general practice, geriatrics, internal medicine, general surgery, oncology, and psychiatry) were asked to participate (i.e., the same specialities as in the 2007 study). Furthermore, all registered palliative care physicians in Sweden (n = 127) were invited to participate. Palliative care started as a specialty in Sweden in 2015, so this group was not included in the 2007 study.

### Acquiring physicians’ postal addresses and clinical specialties

Random samples of the seven specialties and postal addresses were requested from a commercial database, IQVIA in Stockholm. Some letters were returned to sender due to unknown addressee (n = 50), and these questionnaires were omitted for the purpose of calculating the response rate. Table 1 presents statistics about the respondents. The response rate differed somewhat among the different clinical specialties, which might be because the database from which names were randomly selected might not have been entirely accurate about the actual main clinical specialty. A number of respondents (n = 121) listed more than one specialty and in these cases, the first specialty listed was registered as the primary specialty. Sixteen respondents listed their first specialty as something other than one of the seven mentioned specialities, and these respondents were classified as ‘other’ clinical specialties.

**Table 1:** The clinical specialities, number of possible responders for each speciality, response rate, sex distribution, and median age

Specialty + numbers	Response rate (%)	Sex (M/F) (%)	Median age (min–max) (years)
Psychiatrists (n = 240)	52.9	47.2/52.8	58 (31–80)
Surgeons (n = 241)	57.3	71.7/28.3	48 (27–79)
GPs (n = 243)	58.0	51.1/48.9	47 (27–74)
Oncologists (n = 244)	59.4	43.4/57.2	48 (28–78)
Internists (n = 243)	66.3	61.5/38.5	48 (28–79)
Geriatricians (n = 243)	53.1	34.1/65.9	51.5 (29–72)
Palliativists (n = 123)	61.0	33.3/66.7	56 (37–83)
Others (n = 15)		9/6	55 (28–73)
Totally (n = 1577)	59.2	50.5/49.5	50 (27–83)

*GPs* general practitioners, *n* number of respondents

### Questionnaire

The postal questionnaire was commissioned by the Swedish Medical Society in collaboration with Karolinska Institute in Stockholm and was distributed in the autumn of 2020. The first questionnaire was followed by a reminder letter mailed ten days later. Another ten days later, if no response had been received, the questionnaire was posted again. Finally, after yet another ten days, a short version of the questionnaire (including only two key questions and background variables) was sent to those who had still not responded.

The questionnaire entailed twelve items about PAS (see Box 1 for definitions of this term and its distinction from euthanasia). The first question regarded the respondent’s main attitude towards PAS given certain conditions. The subsequent questions were about the participants’ own attitudes about the possibility of receiving legal PAS at the end of their own lives, and about prescribing medication for self-administration to a competent patient who requested the drugs. The response options were *Yes*, *No* and *Undecided,* in order to be identical to the earlier survey from 2007. Space was left for free comments after each item, but in the present paper we focus on the primary, quantitative results. The cover letter with its invitation to participate and information for the participants about the study and the applied terminology used are also provided (see Box 1).

The short version of the questionnaire included only two questions: first, what was the respondent’s attitude towards PAS, and second, what would happen to the respondent’s own trust in healthcare if PAS was legalized and background variables.

Respondents to the long questionnaire were also asked to justify their responses to the three main questions by being asked to choose among various fixed and optional arguments for or against PAS. The principles underlying these arguments were patient autonomy or non-maleficence. Furthermore, respondents were asked to prioritize those responses according to which argument(s) they personally thought most persuasive.

The questionnaire also included questions about what physicians thought would happen to patients’ trust in healthcare if PAS were legalized in Sweden, and whether the respondent’s own trust in healthcare would be affected. If a respondent thought that their own trust in healthcare would decrease, that response was interpreted as a negative attitude about PAS, and if trust in healthcare would increase, that response was interpreted as a positive attitude. Finally, respondents were asked about their age, sex and clinical specialty, and were encouraged to write general comments.

Overall, the questionnaire was similar to the one used in the previous study in 2007, which we wished to match as closely as possible so that the two studies could be easily and usefully compared. Revisions include changes to the main question about attitude, i.e., a few clarifying words were added to the fifth question about decision-making competence, and unbearable suffering was excluded as a necessary condition. In addition, questions about readiness to prescribe drugs for PAS and/or having the possibility to be offered PAS were added, while a few questions about ethical arguments were removed [11].

### Statistical analysis

The responses were registered and analysed using the EPI-info 6.04 software program [15]. When calculating proportions, 95% confidence intervals (95% CI) was applied, assuming that a non-overlapping 95% CI equals a hypothesis test having a *p*-value < 0.05. All methods were performed in accordance with the relevant guidelines and regulations.

### Ethical review

The study was approved by the Swedish Ethical Review Authority, Dnr: 2020–01,842, and no separate informed consent document was required.

## Results

### Participants

The sample population comprises 1 577 possible respondent physicians. Of these, 934 responded, and 819 answered the long version of the questionnaire and 115 answered the short version, for an overall response rate of 59.2% (95% CI 56.0–62.4). Background variables are presented in Table 1.

The sex distribution of the respondents was the same as the overall distribution within these specialties (as listed by the Swedish Medical Association, 13).

### Main outcome

Combining the six specialties, 47.1% (95% CI 43.5–50.7) of respondents would accept PAS, 33.2% (95% CI 29.8–36.6) would not accept PAS, and 19.7% (95% CI 16.8–22.6) were *Undecided* about PAS. In the 2007 study, 34.9% (95% CI 31.5–38.3) of respondents in those six specialties said they would accept PAS, 39.7% (95% CI 36.2–43.2) would not accept PAS, and 25.4% (95% CI 22.3–28.5) were *Undecided*. Thus, compared to 2007 study, attitudes in 2020 were significantly more accepting towards PAS.

Among palliativists, 26.3% (95% CI 16.4–36.2) would accept PAS, 55.3% (95% CI 44.1–66.5) would not accept PAS, and 18.4% (95% CI 9.7–27.1) were undecided.

**Fig. 1:**
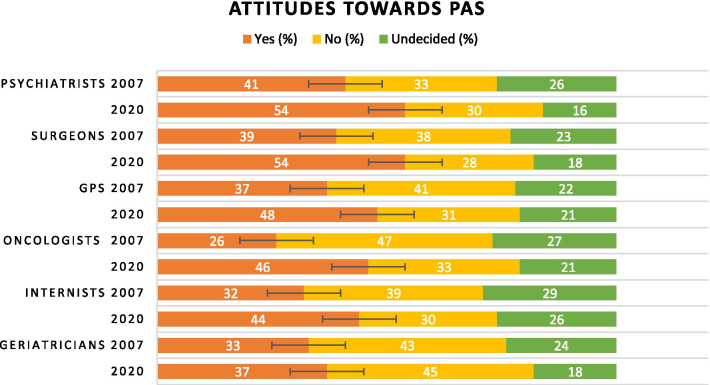
Comparison of attitudes toward PAS in the 2007 and 2020 studies as reflected by the proportions of each specialty who responded *Yes*, *No,* or *Undecided*. Palliative medicine was not its own specialty until 2015. The horizontal black lines between *Yes* and *No* are the 95% confidence intervals. *GPS* general practitioners

Respondents from all specialties seem to have changed their general attitudes towards acceptance of PAS (Fig. 1). Oncologists seem to have changed most significantly, from 26% accepting PAS (95% CI 19–33) in 2007 to 46% (95% CI 38–54) in 2020. Surgeons and psychiatrists, who were most accepting of PAS in the 2007 study, were even more accepting in the 2020 study. Geriatricians were the only group whose responses did not reflect any statistically significant change in attitude. The general tendency was that physicians from all the six specialties tended to be more decisive, and accordingly, there were fewer who reported being undecided.

Regarding age groups, among the compared six specialties and the principal attitude, younger physicians (< 46 years) were significant more accepting towards PAS [53.9% (95% CI 48.1–59.7)] than middle aged physicians (46–60 years) [40.2% (95% CI 36.2–46.2)] (see Table 2).

**Table 2:** The age groups and the three questions regarding (1) the principal attitude towards PAS; (2) whether I would like to have PAS myself; (3) whether I would consider prescribing the drugs needed for PAS

Principal attitudes	Response options		
	Yes (%)	No (%)	Undecided (%)
Age < 46 years (n = 284)	53.6% (48.1–59.7)	26.1% (21.0–31.2	20.0% (15.4–24.6)
Age 46–60 years (n = 259)	40.2% (34.2–46.2)	36.7% (30.8–42.6)	23.2% (18.1–28.3)
Age > 61 (n = 185)	47.1% (40.0–54.2)	37.0% (30.8–43.9)	13.8% (8.9–18.7)
Would have PAS myself			
Age < 46 years (n = 283)	48.0% (42.2–53.8)	26.9% (21.7–32.1)	25.1% (20.0–30.2)
Age 46–60 years (n = 259)	39.4% (33.4–45.4)	35.9% (30.1–41.7)	24.7% (19.4–30.0)
Age > 61 years (n = 185)	44.9% (37.7–52.1)	36.7% (29.8–43.6)	18.4% (12.8–24.0)
Would prescribe drugs			
Age < 46 years (n = 284)	37.0% (31.4–41.6)	39.1% (33.4–44.8)	23.9% (18.9–28.9)
Age 46–60 years (n = 258)	30.2% (24.6–35.8)	50.0% (43.9–56.1)	19.8% (14.9–24.7)
Age > 61 years (n = 185)	36.8% (29.8–43.6)	48.1% (40.9–55.3)	15.1% (9.9–20.3)

Response options were *Yes*, *No*, *Undecided*. The results are presented as proportions with a 95% confidence interval (CI)

Table 2 shows that the attitudes of older physicians were closer to younger physicians than to middle-aged physicians, but regarding all three questions, the older physicians tended to be more decisive than the middle aged or the younger ones.

In order to validate the responses about the general attitude towards PAS, we used a control question, namely the hypothetical question about how trust in healthcare would change if PAS were to be legalized, assuming that decreased trust indicated perceived adverse consequences, and increased trust indicated perceived beneficial consequences (Table 3).

**Table 3:** Hypothetical change in trust if PAS was legalized in Sweden sorted by clinical specialty, sex, and age

Specialties	My own trust in healthcare would…		
	Decrease (%)	Not influenced (%)	Increase (%)
Psychiatrists (n = 122)	29.5 (21.4–37.6)	48.4 (39.5–57.3)	22.0 (14.6–29.4)
Surgeons (n = 134)	21.6 (14.6–28.6)	62.0 (53.8–70.2)	16.4 (10.1–16.4)
GPs (n = 139)	30.2 (22.6–37.8)	56.8 (48.6–65.0)	13.0 (7.5–18.6)
Oncologists (n = 141)	40.4 (32.3–48.5)	51.8 (43.6–60.0)	7.8 (3.4–12.2)
Internists (n = 158)	29.7 (22.6–36.8)	57.0 (49.3–64.7)	13.3 (8.0–18.6)
Geriatricians (n = 124)	46.0 (37.2–54.8)	37.9 (29.4–46.4)	16.1 (9.6–21.6))
Palliativists (n = 72)	65.3 (54.3–76.3)	26.4 (16.2–36.6)	8.3 (1.9–14.7)
Other specialties (n = 16)	6/16	5/16	5/16
Totally (n = 906)	35.4 (32.4–38.4)	50.2 (46.9–53.7)	14.4 (12.1–16.7)
Sex			
Males (n = 458)	27.7 (23.6–31.8)	55.8 (51.3–50.3)	16.5 (13.1–19.9)
Females (n = 448)	38.6 (34.1–43.1)	47.8 (43.2–52.4)	13.6 (10.4–16.8)
Age			
< 46 year (n = 328)	27.7 (22.9–35.5)	55.8 (50.4–61.2)	16.5 (12.5–20.5)
46–60 year (n = 343)	39.1 (33.9–44.3)	49.3 (44.0–54.6)	11.6 (8.2–15.0)
> 61 year (n = 231)	40.2 (33.9–46.5)	44.6 (38.2–51.0)	15.2 (10.6–21.8)

*GPs* general practitioners, *n* number of respondents; 95% confidence intervals indicated in brackets

A general tendency was that a majority of the respondents from the six specialties stated that their own trust in health care would not be influenced if PAS were legalized. If these respondents are counted together with those whose stated that their trust would increase, we can infer that 67.1% (95% CI 63.9–70.3) probably would not actively fight against PAS legalization. The corresponding result from the 2007 survey was 39.5% (95% CI 35.7–43.5). As contrast to these results, a majority of palliativists [65.3% (95% CI 54.3–76.3)] stated that their own trust in healthcare would decrease if PAS were legalized.

Among those who stated that their own trust in healthcare would not be influenced (n = 456), 59.9% in principle accepted PAS, 15.8% were against PAS, and 24.3% were undecided.

The questionnaire also included questions about whether or not the respondents would like to be offered PAS for themselves, and whether or not they would consider prescribing the drugs for self-administration to an eligible patient who requested it. As can be seen from Table 4, fewer respondents were in favour of PAS when the question changed from somewhat theoretical (about general attitude) to empirical (about how a physician would behave in a real situation). Moreover, when the question changes from ‘general attitude’ toward ‘actual prescription’, respondents from all specialties tended merely to say no (Table 4).

**Table 4:** Attitudes toward PAS, whether respondents would like to have PAS as an option, and whether respondents would consider prescribing such drugs for self-administration in relation to how the participants’ own trust in healthcare would be affected

Specialties	Principal attitude yes (%)	Would have PAS myself: yes (%)	Would consider prescribing drugs: yes (%)
Psychiatrists (n = 127/109/108)	54.3 (45.6–63.0)	50.5 (41.1–59.9)	40.7 (31.4–50.0)
Surgeons (n = 138/128/127)	54.3 (46.0–62.6)	55.5 (46.9–64.1)	39.4 (30.9–47.9)
GPs (n = 141/116/116)	48.2 (40.0–56.4)	44.8 (358.8–53.8)	37.9 (29.1–46.7)
Oncologists (n = 145/127/127)	45.5 (37.4–53.6)	36.2 (27.8–44.6)	31.5 (23.4–39.6)
Internists (n = 161/144/145)	43.5 (35.8–51.2)	41.0 (33.0–49.0)	34.5 (26.8–42.2)
Geriatricians (n = 129/112/112)	37.2 (28.9–45.5)	34.8 (26.0–43.6)	21.4 (13.8–29.0)
Palliativists (n = 76/70/70)	26.3 (16.4–36.2)	27.1 (16.7–37.5)	24.3 (14.3–34.3)
Others (16/13/13)	7/16	7/13	3/13
Totally (n = 819/818/818)	45.4 (42.0–48.8)	42.5 (39.1–45.9)	31.1 (27.9–34.3)

The results are presented as proportions of those who answered *Yes* among all clinical specialties with a 95% confidence interval (in brackets). General practitioners = GPs; n = number of respondents per column

In total, 35.3% (95% CI 32.0–38.6) answered *No* regarding the principle attitude and 19.3% (95% CI 16.6–22.0) answered *Undecided*. Regarding having PAS for oneself, 34.8% (95% CI 31.5–38.1) answered *No* and 22.6% (95% CI 19.7–25.5) were *Undecided*; regarding considering prescribing drugs to an eligible patient, 47.4% (95% CI 44.0–50.8) answered *No* and 19.1% (95% CI 16.4–21.8) were *Undecided*.

If we exclude the palliativists and focus on the specialties that were included in the 2007 study, the proportion who were accepting of PAS in principle (that is, responded *Yes*) was 47.1% (95% CI 43.5–50.7), 33.2% responded *No*, and 19.7% were *Undecided*. The answers followed a similar trend in to the question about whether or not they would like to have the option for PAS for themselves; 43.8% (95% CI 40.4–48.2) answered *Yes*, 32.7% (95% CI 29.3–36.1) answered *No* and 23.5% were *Undecided*. When asked if they would prescribe drugs for PAS, 34.3% (95% CI 30.9–37.7) answered *Yes*, 45.6% (95% CI 42.0–49.2) answered *No*, and 20.1% were *Undecided*.

We have examined the association between the question “What would happen to [the respondent’s] own trust in healthcare if PAS was legalized?” in terms of the two collapsed response options *Increase* or *Decrease* (excluding the response option *Not be influenced*) and the response option *Yes* or *No* to PAS (excluding the response option *Undecided*). The Risk Ratio (RR) regarding the principal attitude towards PAS was 16.7 (95% CI 10.2–27.2); for having the option for PAS for oneself, RR was 20.2 (95% CI 11.3–35.9); finally, for prescribing the drugs, RR was 253.6 (95% CI 35.9–1793.7).

In the questionnaires, the respondents were asked to prioritize a number of fixed and optional arguments for or against PAS. Fixed arguments in favour of PAS focused on autonomy and whether respect for a patient’s autonomy should overrule the non-maleficence principle. Fixed arguments against PAS were that the non-maleficence principle should overrule concerns about autonomy, and that a patient in such a situation did not know their own best interests. The results are presented in Table 5.

**Table 5:** Main arguments for and against PAS when asked which arguments were the most important, and put in the context of whether the respondent’s own trust in healthcare would *Increase*, *Decrease*, or *Not be influenced* if PAS was legalized, presented as proportions (95% confidence intervals in brackets)

Own trust would:	Arguments for and against PAS		
	Autonomy based	Non-maleficence based	Other
Decrease (%) (n = 241)	6.6 (3.5–9.7)	76.4 (71.0–81.8)	17.0 (12.3–21.7)
Not be influenced (n = 343)	65.0 (60.0–70.0)	26.8 (22.1–31.5)	8.2 (5.3–11.1)
Increase (n = 106)	91.5 (81.2–96.8)	0.9 (0.0–2.7)	7.6 (2.6–12.6)

Autonomy-based means that a patient’s autonomy is respected rather than protected. Non-maleficence-based means that a patient’s autonomy is protected rather than respected. A large number of respondents (n = 224) abstained from prioritizing these arguments

Younger physicians (< 46 years) tended to prioritize autonomy arguments [52.9% (95% CI 47.0–58.8)] more than middle-aged respondents (46–60 years old), who prioritized autonomy arguments to a lesser extent [41.1% (95% CI 36.4–47.2] in favour of the non-maleficence arguments. The oldest group of physicians (> 61 years) responded in a way similar to the youngest group [52.2% (95% CI 44.9–59.5)]. The younger physicians tended, to a lesser extent, to favour the non-maleficence argument [36.4% (95% CI 30.8–42.0)] more than the middle aged physicians [44.8% (95% CI 38.6–51.0)] (Chi-2 = 7.3, df = 2 and p = 0.03).

## Discussion

Compared to the 2007 survey, significantly higher proportions of six clinical specialists accepted PAS, and fewer could not accept PAS or were undecided about it. This shift in attitude is in line with follow-up studies from other countries [1–6]. There might be several reasons for the general change in attitudes. First, younger physicians (< 46 years old) reported a more accepting attitude in the 2020 study, at least compared to middle aged (46–60 years old) physicians, which may be explained by extended teaching in medical school as well as more training in the process of shared decision-making in clinical practice, including end-of-life care [16]. Moreover, a patient law was introduced in Sweden in 2015 that gives patients the right to participate in the medical decision-making process [17]. Even though the law by itself did not require any changes to day-to-day clinical practice, the law-making process was a step in the direction of emphasis on patients’ rights.

Interestingly, the younger (< 46 years) and the older (> 61 years) physicians both seemed to prioritize the autonomy argument, whereas the age group in the middle (between 46–60 years) prioritized the non-maleficence argument.

Some other differences between the results from the 2007 survey and the 2020 study are worth noting. For instance, oncologists changed their views the most, moving from opposing to supporting PAS. This trend among Swedish oncologists is in accordance with the general trend among Swedish doctors, but seems to be against the trend in attitudes towards PAS among oncologists worldwide [18]. A possible explanation for the Swedish trend might be that palliative care has become its own clinical specialty, and many palliative care physicians come from the speciality of oncology. A majority of palliative care physicians have a negative attitude towards PAS (Table 3), and there is a good chance that some of these physicians were part of the random sampling of oncologists in the 2007 survey. However, it is more difficult to explain why internists, surgeons, and psychiatrists seem to have more accepting attitudes about PAS in 2020 than they did in 2007. It is possible that a new generation of physicians is simply stressing a patient’s right to participate in decision-making. The more recent medical students have probably also been taught that if patients are not allowed to share in decision-making at the end of life, strong arguments are needed to justify that position (Box 1). Moreover, a study about deep continuous sedation showed that palliativists were currently more inclined to initiate such sedation on patient’s request [18].

**Box 1:** Definition of physician assisted suicide (PAS) and euthanasia

PAS means that a patient who is found to be competent and fulfils certain criteria, and who visits a physician and requests prescription of drugs by which the patient might commit suicide in order to put an end to life, or prevent unbearable suffering at the end of life. The patient is supposed to be able to take the drugs on her or his own, meaning that the physician’s role is only to prescribe the drugs. The patient might use the drugs or abstain from taking the drugs, if they decide the suffering is bearable	
Euthanasia means that a physician injects lethal doses of a drug(s) upon a competent patient’s request. The physician’s role is active and the patient will die immediately after having received the injection or drip. The criteria for euthanasia are similar to those for prescribing life-ending drugs for self-administration	

When comparing the results of a Swedish survey of PAS to similar surveys done in other countries, it is important to know that there is no tradition or legislation in Sweden that allows physicians to conscientiously object to specific issues. For instance, when abortion rights were introduced in Sweden in 1975, it meant that all gynaecologists were required to perform abortions [14]. The fact that ‘conscientious objector’ is not a recognized status means that physicians in Sweden may think that if PAS is legalized, all physicians would be required to be involved and prescribe the drugs. Under these conditions, Swedish physicians might be more hesitant to answer (or think) positively about PAS compared to physicians from other countries with traditions of conscientious objection. Nevertheless, a recent large-scale British survey about prescribing PAS drugs for self-administration showed results comparable to ours: 50% of the respondents supported legalizing PAS, 39% opposed legalisation, and 11% were indecisive [1]. The trend in the present study seems to be in accordance with the trends seen in other countries with no assisted suicide legalization [1–6].

Perhaps not surprisingly, palliative care physicians were mostly against PAS, which is also in accordance with position papers in the field [19–21], although even here approximately a quarter of palliative physicians supported PAS.

### Strengths and limitations

This study has a number of strengths. First, we believe that our random sample is truly representative of the population of the clinical specialties, because the sex distributions in each of the clinical specialities (which is known) corresponded to the distribution of our participants, which strengthens our assumption that this sample is representative [22]. Second, because the present study was conducted in the same manner as the 2007 survey, the two surveys are comparable to each other, allowing us to discern trends. The differences between the two surveys were quite small: the 2020 version added questions and presented fewer fixed reasons for accepting or not accepting PAS in order to shorten the time required to respond. Some formulations have also been changed, but in order to avoid euphemisms and dysphemisms that could frame the questionnaire and make it biased [23], we used the same terminology in the cover letter, where it is clear that the questionnaire is about PAS. We have also used the same value-laden terms such as “commit suicide” in both the 2007 study and the 2020 study. The present study also included the same questions regarding what would happen to the participant’s own trust in healthcare if PAS was legalized in Sweden. The strong association between attitude towards PAS and the respondent’s anticipated own trust in healthcare (were PAS to be legalized) indicates that there was a good correlation between the *Yes* and *No* answers, and whether trust would increase or decrease, or not change.

A factor that is both a strength and a limitation is that respondents were allowed only three alternative answers (*Yes*, *No*, *Undecided*) to questions about attitudes to PAS. This decision was made for the 2007 study, and the 2020 version used the same three possible answers in order to correspond directly to the earlier survey. However, this constraint may have created a higher degree of polarization than if there had been broader spectrum of more nuanced answers.

A limitation of the study is that we had a rather low response rate (59.2%) compared to the 2007 survey (75%). One reason might be that the 2020 survey was conducted during an ongoing and second peak of the Covid-19 pandemic (between the beginning of November and the end of December 2020), putting strain on the respondents in their professional capacities. There were, however, no differences in response patterns among those who responded to the first (n = 600) or second (n = 219) dispatches (the long questionnaire) or the third (n = 115) dispatch (the short questionnaire) in regards to the main issue of attitude towards PAS. It seems reasonable to assume that responses of those who did not participate would have been similar to those who did.

We do not believe that the slight changes to the 2020 version of questions about reasons for being for or against PAS caused fewer physicians to respond to the long version than did in 2007. If these questions had been perceived as particularly difficult to understand or to respond to, then we might have expected more physicians to simply [wait for and] answer the short version of the questionnaire. In fact, significantly fewer physicians answered the short version of the questionnaire in 2020 than answered the short version in 2007 (p = 0.003). This difference in response rate can be taken as at least weak evidence that the long questionnaire in 2020 was not perceived as more difficult than the 2007 version.

Finally, we are aware that the wording and framing of a questionnaire, as well as the introduction with preconditions in the cover letter, might influence how the participants respond, at least regarding the general public [10, 23]. Some of the preconditions themselves might have been subject to different interpretations, such as “the patient is at the end of life”, but we used the same phrase that was used in the 2007 study. In the present study, we assume that physicians might also be influenced by framing effects, so for instance, referring to a patient’s autonomy might not be quite clear: does ‘respect for patient autonomy’ mean ‘respect for a patient’s right to participate in decision-making’, or does it mean that ‘a patient must be protected if he/she is not able to make decisions in his/her own best interest’? When we referred to the autonomy principle, we meant it as respecting a competent patient’s right to participate in decisions, but that might not have been every respondent’s understanding. At the same time, while conceding the potential for phrases like ‘committing suicide’ to frame and influence the results, we would expect that the framing effect in 2007 would have been the same as in the 2020 study, and therefore the results are still comparable.

## Conclusions

On average, about 47% of respondents reported an accepting attitude towards PAS in Sweden. Thirty-three percent were prepared to prescribe the needed drugs under the condition of legal possibility.

Compared to the 2007 survey, the present study indicates a significantly higher acceptance of PAS today under the stipulated conditions. Moreover, a large majority of physicians stated that their trust in healthcare would neither decrease nor be influenced at all if PAS was legalized, which also points to a trend of a more accepting attitude towards PAS.

In general, compared to middle aged physicians (46–60 years), both younger physicians (< 46) and older physicians (> 61 years) seem to support PAS based on respect for a patient’s right to participate in decision-making at the end of life. If this trend persists, we might in the future see an increased willingness among Swedish physicians to participate in PAS.

## Data Availability

All data are available on request, please contact the corresponding author.
